# Survey of perceptions and educational needs of primary care providers regarding management of patients with class II and III obesity in Ontario, Canada

**DOI:** 10.1186/s12875-020-01356-x

**Published:** 2021-01-09

**Authors:** Boris Zevin, Mary Martin, Nancy Dalgarno, Linda Chan, Nardhana Sivapalan, Robyn Houlden, Richard Birtwhistle, Karen Smith, David Barber

**Affiliations:** 1 Department of Surgery, Queen’s University, Kingston, ON Canada; 2 Department of Family Medicine, Queen’s University, Kingston, ON Canada; 3 Office of Professional Development and Educational Scholarship, Queen’s University, Kingston, ON Canada; 4 Department of Medicine, Queen’s University, Kingston, ON Canada; 5 Department of Physical Medicine and Rehabilitation, Queen’s University, Kingston, ON Canada

**Keywords:** Primary care, Obesity management, Bariatric surgery, Medical weight loss, Survey study

## Abstract

**Background:**

Primary care providers (PCPs) are typically the primary contact for patients with obesity seeking medical and surgical weight loss interventions; however, previous studies suggest that fewer than 7% of eligible adult patients are referred to publically funded medical and surgical weight loss interventions (MSWLI).

**Methods:**

We performed an anonymous survey study between October 2017 and June 2018 to explore the knowledge, experiences, perceptions, and educational needs of PCPs in Southeastern Ontario in managing patients with class II and III obesity.

**Results:**

Surveys were distributed to 591 PCPs (*n* = 538 family physicians; *n* = 53 nurse practitioners) identified as practicing in the Southeastern Ontario and 92 (15.6%) participated. PCPs serving a rural population estimated that 14.2 ± 10.9% of patients would qualify for MSWLI compared to 9.9 ± 8.5% of patients of PCPs serving an urban population (*p =* .049). Overall, 57.5% of respondents did not feel competent prescribing MSWLI to patients with class II/III obesity, while 69.8% stated they had ‘good’ knowledge of the referral criteria for MSWLI. 22.2% of respondents were hesitant to refer patients for bariatric surgery (BS) due to concerns about postoperative surgical complications and risks associated with surgery. Only 25% of respondents were comfortable providing long-term follow up after BS, and only 39.1% had participated in continuing education on management of patients with class II/III obesity in the past 5 years.

**Conclusion:**

The majority of PCPs believe there is a need for additional education about MSWLI for patients with class II/III obesity. Future studies are needed to develop and compare the effectiveness of additional education and professional development around risks of contemporary BS, indications to consider referral for MSWLI, management and long-term follow-up of patients after BS.

**Supplementary Information:**

The online version contains supplementary material available at 10.1186/s12875-020-01356-x.

## Background

Obesity is a chronic disease associated with reduced quality of life and comorbidities that contribute to increased health care costs and a significant reduction in life expectancy [1, 2]. Sustained weight loss in patients with obesity is associated with prevention, alleviation, and resolution of obesity-related comorbidities [3]. Primary Care Providers (PCPs) are well-positioned to direct timely treatment and management of obesity in their patients as they are the primary contact for patients seeking medical and surgical weight loss interventions (MSWLI). Bariatric surgery (BS) is the only intervention that results in significant and sustained weight-loss, improvement and/or resolution of obesity-related comorbidities in patients with class II and III obesity (Body Mass Index (BMI) 35.0–39.9 kg/m^2^ and BMI ≥ 40.0 kg/m^2^, respectively) [1, 4–10]; however, PCPs continue to prescribe lifestyle modification as the recommended treatment for these patients [11]. Unfortunately, fewer than 5% of patients with class II and III obesity are successful in achieving sustained weight reduction with lifestyle modification alone [12–15]. In 2018, an estimated 7.3 million Canadian adults were classified as having class I obesity or greater (BMI ≥ 30.0 kg/m^2^), and continued increases have been noted in the prevalence of class II and III obesity from 1.207 million in 2009 to 1.774 million in 2017 [16]. Millions of Canadians continue to struggle with the obesity-related co-morbidities and social issues associated with obesity.

In Ontario, any individual with Class II obesity and an obesity-related comorbidity, or class III obesity is eligible for referral to a Bariatric Centre of Excellence by their PCP to be assessed for publicly funded MSWLI. However, access to MSWLI interventions in Ontario remains poor with fewer than 7% of all eligible adult patients being referred [17] and only 1% actually undergoing these potentially life-saving interventions [16]. As such, there remains a gap between evidence-based care and actual practice of PCPs in the management of patients with class II and III obesity.

This study explores the knowledge, experiences, perceptions, and educational needs of PCPs in Southeastern Ontario in managing patients with class II and III obesity.

## Methods

A survey methodology was our study design and it was approved by Queen’s University and Affiliated Hospitals Health Sciences Research Ethics Board (#6021335**)**.

### Setting

This study was conducted between October 2017 and June 2018 in the Southeast Local Health Integration Network (SELHIN) in Ontario, Canada. The SELHIN covers approximately 25,000 km^2^ and has a population of 500,000 (3.6% of the Ontario population) [18]. Twenty-five percent of the population live in an urban centre, while 45% live in rural areas [18].

### Participants and recruitment

We obtained a list of all PCPs (family physicians and nurse practitioners) practising in the SELHIN by searching postal codes in a publicly available online database (College of Physicians and Surgeons of Ontario, https://www.cpso.on.ca/). Using a Dillman design [19], an invitation to participate was sent via email where possible, with hard copies faxed and/or mailed to offices where not. Hard copies were also distributed at local continuing professional development (CPD) sessions attended by PCPs. Two reminder emails were sent out, where possible, to improve recruitment. PCPs were eligible to participate if they were currently practicing, could communicate in English and signed the enclosed consent form (hard copy) or indicated consent on the online survey form. Participants also had the option to enter an iPad draw.

### Survey design

The survey consisted of 25 items organized into six parts: Plans of Care for Patients with class II and III obesity; Experiences with BS; Reservations about BS; Future Treatment of Severe Obesity; CPD and Demographics (Additional file 1). It took participants 15 min to complete. Survey items included open-text, Likert scales, and yes/no answers with branching questioning to reduce the number/complexity of responses. Questions were constructed based on a literature review and consensus among researchers. It was pilot-tested with a PCP and member of the research team [DB] through a think aloud [20, 21] and then revised [BZ, ND, MM, NS, LC]. The electronic version of the survey was constructed in Qualtrics [22] [MM], had a completeness check, back button and was piloted by three non-authors with experience in developing online surveys. To comply with ethical standards, participants could choose to skip any questions they wished not to answer.

### Data collection

Data was collected anonymously via an open online survey, which was available to anyone who had the link. Hard-copy survey responses were entered into the online survey for data analysis.

### Analysis

Only submitted (completed) online surveys were included in analysis. As participants could choose to skip survey questions, proportions were calculated based on number of non-blank responses received per question. Statistical analyses were conducted using SPSS version 24 [23] through descriptive statistics, and independent sample t-tests used to identify significant compare groups based on demographic variables (2-sided, α = 0.05). For likert scale questions, responses were dichotomized into agree or disagree groups for comparisons. A Pearson’s Correlation Coefficient was used to examine for correlation between demographic variables.

## Results

Surveys were distributed to 591 PCPs (*n* = 538 family physicians; *n* = 53 nurse practitioners). Of these, 92 (15.6%) completed the survey. Survey completion rate (proportion of eligible participants that completed the survey out of all who consented to participate) was 89.3% (92/103). The demographic characteristics are shown in Table 1, including counts for non-responders. Where applicable, values are presented as mean ± standard deviation (SD).

**Table 1:** Demographics of Survey Respondents

Characteristic	No. of respondents (%)
**Profession**	
Family Physician	84 (91.3)
Nurse Practitioner	6 (6.5)
No answer/prefer not to answer^a^	2 (2.2)
**Gender**	
Male	30 (32.6)
Female	57 (62.0)
No answer/prefer not to answer	5 (5.4)
**Years in Practice**	
0–10	36 (39.1)
11–20	12 (13.0)
21–30	21 (22.8)
31+	17 (18.5)
No answer/prefer not to answer	6 (6.5)
Mean (± SD)	17.7 (± 13.4)
**Practice Types**	
Solo Practice	3 (3.3)
Group Practice^b^	31 (33.7)
Family Health Team	35 (38.0)
Other	17 (18.5)
No answer/prefer not to answer	6 (6.5)
**Interprofessional Practice**	
Yes	63 (68.5)
No	24 (26.1)
No answer/prefer not to answer	5 (5.4)
**Population Served**	
Urban	51 (55.4)
Rural	34 (37.0)
No answer/prefer not to answer	7 (7.6)

^a^Demographic questions were optional in the survey to preserve anonymity

^b^Not including Family Health Team

Columns may not add up to 100% due to rounding

Male participants were significantly older than females (mean age 54.2 ± 12.6 vs. 44.0 ± 11.7 years, *p <* .001), and had been in practice significantly longer (25.21 ± 13.5 vs. 13.9 ± 11.7 years, *p* < .001). PCPs in a non-interprofessional practice (IPP) were significantly older than those in an IPP (53.9 ± 13.1 vs. 45.0 ± 12.1 years, *p* = .004). Similarly, those in a non-IPP had been in practice significantly longer compared to those in an IPP (23.6 ± 13.6 vs. 15.5 ± 12.7 years, *p* = .012).

### Plans of Care for Patients with class II and III obesity

Surveyed PCPs estimated that 11.6 ± 9.8% of patients in their practice would qualify for MSWLI. PCPs serving a rural population estimated that 14.2 ± 10.9% of their patients would qualify for MSWLI, which was significantly higher than PCPs serving an urban population (9.9 ± 8.5%, *p =* .049). Overall, 53.3% (49/92) of PCPs were not aware of contemporary international guidelines that recommend considerations of BS for patients with class II/III obesity and type 2 diabetes for BS. Overall, 57.5% of respondents (50/87) did not feel competent prescribing medical weight loss interventions to their patients with class II and III obesity, and 87.1% (74/85) disagreed that they are usually successful in helping patients with class II and III obesity lose weight without BS.

### Referrals for bariatric surgery

**Fig. 1:**
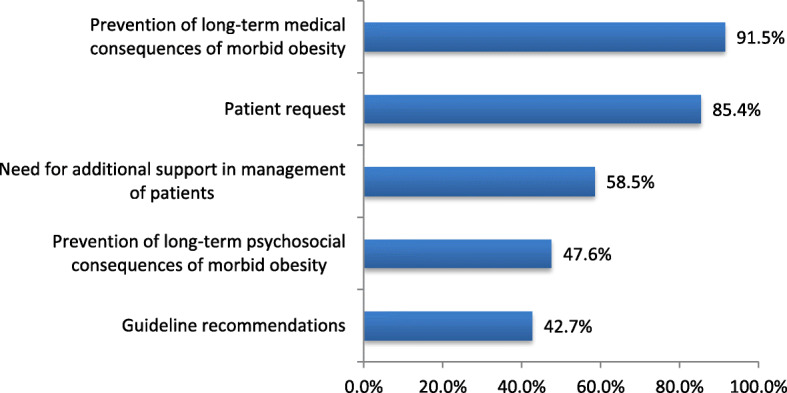
PCPs’ reasons for referring patients for bariatric surgery

Overall, 69.8% (50/86) of respondents agreed they had ‘good’ knowledge of referral criteria for BS. Males (19/30, 63.3%) were significantly more likely to agree that they are aware of the guidelines for BS referral compared to women (21/57, 36.8%) (*p* = .018). Overall 95.4% (82/86) of PCPs have referred patients for BS, however, 60.9% (53/87) reported that they had referred 10% or fewer of their patients who would qualify for BS. Figure 1 summarizes the most frequently cited reasons for referral for BS.

When asked about reasons for BS referral, 37.9% (33/87) of PCPs agreed that patients initiated conversations about BS as a potential treatment for their obesity, whereas 44.8% (39/87) agreed that they most often brought it up. PCPs in practice for 0–10 years (8/27, 29.6%) were significantly less likely to initiate discussions about referral for BS than PCPs practicing for 11–20 (7/7, 100%, *p =* .002), 21–30 (14/20, 70%, *p* = .016), and 31+ years (10/13, 76.9%, *p* = 0.013). Additionally, there was a significant weak positive correlation between age of the PCP and likelihood of initiating discussion about referral for BS (r = 0.363. *p* = .003). 61.2% (52/85) of respondents agreed that patients with obesity often seek consultation with them for the purpose of receiving information about BS.

### Reservations about bariatric surgery

**Fig. 2:**
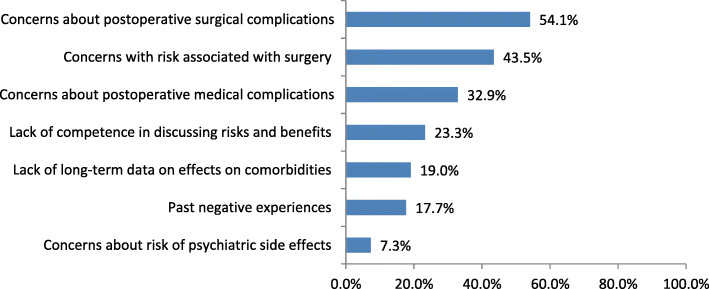
PCPs’ reasons for reservations about referring patients for bariatric surgery

Overall, 22.2% (19/85) of study participants agreed that they are hesitant to refer patients for BS, with the two most common reasons being concerns about postoperative surgical complications and risks associated with surgery. Figure 2 identifies the specific reservations of PCPs about referring their patients for BS.

PCPs serving an urban population were significantly more likely to report that they had reservations about referring their patients for BS due to the lack of long-term data on the effects of surgery on obesity-related comorbidities as compared to those serving a rural population (39.4% (13/33) vs. 11.5% (3/26), *p =* .012). Female PCPs were significantly more likely to report that they had reservations about referring for BS due to past negative experiences as compared to male PCPs (22.7% (10/44) vs. 6.7% (1/15), *p* = .037).

### Perceptions of follow-up after bariatric surgery

Nearly every PCP surveyed (97.7%, 84/86) agreed that long-term follow-up is required after BS; 37.9% (32/87) felt that follow up should be the responsibility of the bariatric surgeon, 25.0% (21/84) were comfortable providing long-term follow up themselves, and 25.6% (21/82) reported having resources necessary to provide good-quality long-term follow-up after BS. Only 18.4% (16/87) of PCPs felt competent in addressing medical complications that may arise after BS.

### Future treatment of class II and III obesity

Participants were asked to rate their level of agreement with the importance of three different approaches to future treatments for patients with class II and III obesity. Overall, 58.8% of PCPs (50/85) agreed that the future treatments must be based primarily on lifestyle intervention and behavioural modification; 43.4% (36/83) believed that it should be based primarily on BS with behavioural and dietary modifications; and 34.5% (29/84) believed it should be based on medical management with dietary restriction. Several differences between groups are summarized in Table 2.

**Table 2:** Differences among respondents about future treatments of patients with class II and III obesity

Future treatment of patients class II and III obesity must be primary based on:	% of PCPs who Agree	Category of PCPs more likely to agree	***p***-value
Lifestyle intervention and behavioural modification	58.8% (50/85)	Interprofessional (vs. non-interprofessional) practice	.05
		Years in practice 11–20 (vs. 31+)	.028
Medical management and dietary restriction	34.5% (29/84)	Interprofessional (vs. non-interprofessional) practice	.043
		Years in practice 0–10 (vs. 31+)	.049
Bariatric surgery with behavioural and dietary modifications	43.4% (36/83)	–	–

### CPD in management of patients with class II and III obesity

Only 39.1% (34/87) of PCPs had participated in education on management of patients with class II and III obesity in the past 5 years. Overwhelmingly, 88.5% (77/87) of PCPs believed there is a need for education on this topic, with PCPs in their first 10 years of practice significantly more likely to agree compared to those who have been in practice for 11–20 years (*p* = 0.033). Overall, 79.3% (69/87) believed that it is very important to be knowledgeable about medical treatment options for obesity; with PCPs serving a rural population significantly more likely to rate this importance higher compared to those serving an urban population (*p* = 0.013). Finally, 63.2% (55/87) believed that it is very important to be knowledgeable about BS for patients with class II and III obesity.

### Interpretation

In this study, we explored the knowledge, experiences, perceptions, and educational needs of PCPs in Southeastern Ontario in managing patients with class II and III obesity. PCPs acknowledged that over 10% of patients in their practice had class II and III obesity and most PCPs agreed that these patients are not likely to succeed in achieving durable weight loss without BS. PCPs in rural locations perceived to have a greater proportion of patients with class II and III obesity as compared to urban locations. These perceptions are in agreement with reported 17.2% of patients with class II and III obesity in primary care in Southeastern Ontario [17]. The perceptions of greater proportion of patients with obesity in rural setting is also in agreement with global findings [24].

Despite an accurate perception of the proportion of patients with class II and III obesity in their practice and the knowledge that BS can prevent long-term medical complications of obesity, more than 60% of PCPs reported referring fewer than 10% of eligible patients for BS. Most PCPs reported initiating the referral for BS following a direct request from a patient, and fewer than half of all PCPs reported being the one initiating conversations about BS with patients. These findings are in agreement with a 2014 Ontario survey [25], which reported that over 70% of physicians have referred no more than 5% of their patients with class II/III obesity for BS. Plausible explanations for the low referral rate include lack of knowledge by PCPs about risks and benefits of contemporary BS, and the role that BS can play in helping patients with obesity improve their quality of life [26].

In our study, more than one third of all PCPs were hesitant to refer patients for BS due to concerns about complications and risks associated with surgery, and medical complications after surgery. In a recent qualitative study of PCPs in Southeastern Ontario, we identified that most PCPs viewed BS as high-risk, with significant short- and long-term post-operative complications [26]. PCPs also viewed BS as a last resort after unsuccessful attempts at all other weight loss interventions [26]. Other studies have highlighted similar concerns of PCPs about associated risks of BS [27–30]. These perceptions are in contradiction with data regarding safety of contemporary BS with an overall complication rate of 11.7% and mortality of 0.16% [31]. In comparison, mortality rate for a cholecystectomy in is nine times higher at 1.36% [32]. This gap in knowledge regarding safety of contemporary BS may contribute to the hesitancy of PCPs to refer their patients, however it can be addressed via ongoing professional development activities.

We identified that PCPs in their first 10 years of practice were significantly less likely to bring up BS with their patients compared to PCPs in all other age groups. We also found a significant positive correlation between age of PCP and likelihood of bringing up BS. This is a surprising finding as BS had been available and covered by a provincial health insurance plan in Ontario for 10 years at the time of this study [33]. Our results do not provide an explanation for this finding; however, similar results have been observed in other studies [25, 34]. PCPs who have been in practice fewer than 10 years may not have been in practice long enough to appreciate the long-term benefits of BS. This finding suggests that greater emphasis should be placed on management of patients with obesity in undergraduate and postgraduate medical education programs for PCPs in order to better prepare trainees for transition to independent practice.

We identified that one in two PCPs were not aware of contemporary guidelines that recommend considerations of BS for patients with class II/III obesity and type 2 diabetes [35, 36], and that one in two PCPs did not feel competent prescribing weight management programs to their patients. Moreover, we also identified a lack of knowledge in PCPs regarding follow-up and care for patients post BS. Our findings are consistent with other literature [25, 37, 38] in that that few PCPs report having the knowledge they need to feel comfortable providing quality aftercare for their patients following BS. These findings are concerning given the potential cost-savings to the health care system by recommending BS to patients with type 2 diabetes and class II/III obesity. Studies from the United States [39] and Europe [40, 41] demonstrate that BS may lead to cost savings to the health care system.

We identified that fewer than half of PCPs in our study region had participated in continuing education on the management of patients with obesity in the past 5 years. The majority of PCPs, however, do believe there is a need for more education about both medical and surgical weight-loss options for patients with class II/III obesity. This was especially true for PCPs in their first 10 years of practice and those in rural locations. However, the most effective pedagogical approach to deliver this professional development is not clear. Lectures and symposia have been shown to have a positive impact on physician knowledge; however, interactive CPD activities that encourage reflection on practice, provide opportunities to practice skills, involve multiple exposures, and are focused on outcomes appear to be the most effective at improving practice and patient health outcomes [42, 43]. Future research should focus on optimizing and evaluating the delivery of CPD activities focused on management of patients with obesity.

We must emphasize that PCPs knowledge, experiences, and perceptions about managing patients with class II/III obesity are a few of the many barriers that patients with obesity face in gaining access to treatments for their chronic disease. Other barriers include insurance policies and funding issues. Many patients are unable to afford the $253.00 per week out-of-pocket cost for the meal replacement required to take part in medical weight loss programs, while Health-Canada approved anti-obesity medications are not covered by any of the provincial/territorial public drug benefit programs [16]. These barriers are system-related, and out of the control of PCPs. Nonetheless, PCPs should be aware of these barriers when managing and helping eligible patients access MSWLI options.

### Study limitations

Though we incentivised participation through offering a draw for an iPad, we observed a relatively low response rate, which was a limitation in this study. As such, this may have led to selection bias, as only those interested in the topic may have been recruited. Furthermore, only 6.5% of participants self-identified as nurse practitioners, further limiting generalizability of our results to the nurse practitioner group. Additionally, the results of the survey used in this study have not been previously validated. Our results are also subject to recall bias, and bias associated with self-reported responses. Additionally, due to the anonymous nature of the study, we did not collect IP addresses and therefore participants may have completed more than one entry. Lastly, our study was conducted in one region of Ontario, which limits generalizability to other contexts.

## Conclusions

We identified a clear need and desire of PCPs for additional education and professional development around risks of contemporary BS, indications to consider referral for MSWLI, management and long-term follow-up of patients after BS. This lack of knowledge regarding BS is likely contributing to the low referral rates for surgery in patients who could benefit greatly from this intervention. Future work should focus on developing and comparing the effectiveness of CPD interventions, especially for PCPs in their first 10 years of practice and those practicing in rural locations.

## Supplementary Information

**Additional file 1.**

## Data Availability

Raw data may be obtained from the corresponding author, Dr. Boris Zevin, upon request.

## Abbreviations

BMI: Body mass index
BS: Bariatric surgery
CPD: Continuing professional development
IPP: Inter-professional practice
MSWLI: Medical and surgical weight loss interventions
PCP: Primary care provider
SD: Standard deviation
SELHIN: Southeast local health integration network
