# Supplementary material for: Survey of perceptions and educational needs of primary care providers regarding management of patients with class II and III obesity in Ontario, Canada
Source: BMC Fam Pract. 2021 Jan 9;22:14. doi: 10.1186/s12875-020-01356-x (PMC7797146; doi:10.1186/s12875-020-01356-x)
Supplement: Supplementary file 1 — Additional file 1. [file 12875_2020_1356_MOESM1_ESM.docx]

**Appendix A**

**Survey Questions**

**Primary Care Provider Survey: Bariatrics**

**Exploring barriers for access to weight management care for morbidly obese patients within the Southeast LHIN**

1. Are you a:
   - Primary Care Physician
   - Nurse Practitioner
   - Other (please specify) ___________________________

**PLEASE NOTE:** For the purpose of this study:

1. ***Obesity*** refers to BMI 30 kg/m2


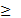

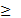


1. ***Morbid obesity*** refers to BMI35 kg/m2.


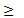

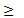


1. ***Patients who meet the criteria for bariatric surgery*** refer to individuals with BMI35 with obesity-related comorbidities (diabetes, hypertension, obstructive sleep apnea, dyslipidemia, asthma, GERD, pulmonary hypertension, depression, anxiety, urinary incontinence, osteoarthritis, etc.) or BMI


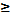

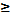

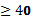

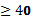


**Part A: Plans of Care for Patients with Morbid Obesity**

1. Which of the following treatments have you recommended to patients? If you have recommended them, how satisfied were you with their outcome?

*(Select all that apply. For those you have recommended, please indicate how satisfied you were with their outcome on the scale provided)*

|  | **Have not recommended** | **IF YOU HAVE RECOMMENDED, how satisfied were you with the outcome?** | | | | | |
| --- | --- | --- | --- | --- | --- | --- | --- |
|  |  | **Very Satisfied** | **Satisfied** | **Neutral** | **Dissatisfied** | **Very Dissatisfied** | **Don’t Know** |
| Drugs |  |  |  |  |  |  |  |
| Diet |  |  |  |  |  |  |  |
| Individual Counseling |  |  |  |  |  |  |  |
| Group Counseling |  |  |  |  |  |  |  |
| Weight loss programs |  |  |  |  |  |  |  |
| Consultations with specialists (e.g., Dietitian, Internal Medicine Physician, Nurse Practitioner) |  |  |  |  |  |  |  |
| Surgery |  |  |  |  |  |  |  |
| Other (please write here) |  |  |  |  |  |  |  |

1. Morbid Obesity:

*(select all that apply)*

- Is easily controlled with dietary changes / exercise alone
- Is difficult to control with dietary changes / exercise alone
- Can be effectively controlled through a medically supervised program
- Cannot be effectively controlled by ANY diet/ exercise program
- Is best treated with surgical intervention

1. Are you aware that international guidelines recommend bariatric surgery to treat type 2 diabetes in appropriate surgical candidates with BMI 40 kg/m2 (BMI 37.5 kg/m2 in Asian Americans), regardless of the level of glycemic control or complexity of glucose-lowering regimens, and in adults with BMI 35.0–39.9 kg/m2 (32.5–37.4 kg/m2 in Asian Americans) when hyperglycemia is inadequately controlled despite lifestyle and optimal medical therapy?


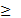

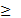

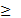

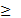


- - Yes
  - No

1. Approximately what percentage of patients with morbid obesity (BMI35 with obesity-related comorbidities or BMI do you have in your current practice?


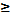

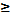

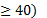

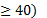


Please write answer:_________________%

**Part B: Experiences with Bariatric Surgery**

1. Approximately how many patients with morbid obesity do you see per month who fit the criteria for bariatric surgery (BMI35 with obesity-related comorbidities or BMI ?


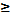

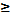

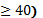

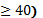


- - 0
  - <5
  - 5-10
  - >10

1. Approximately what percentage of patients with morbid obesity (BMI35 with obesity related comorbitidies or BMI ) in your current practice do you refer for surgical management of their obesity and obesity related comorbidities?


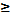

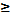

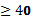

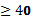


- - 0
  - 1-10%
  - 11-20%
  - 21-30%
  - 31-40%
  - 41-50%
  - 51-60%
  - 61-70%
  - 71-80%
  - 81-90%
  - 91-100%

1. Approximately how many patients with obesity (BMI ≥ 30 kg/m2) do you see per month that fit the criteria for non-surgical weight loss intervention (BMI 30-35 kg/m2)?
   - 0
   - <5
   - 5-10
   - >10
2. Approximately what percentage of patients with obesity (BMI ≥ 30 kg/m2) in your current practice do you **treat or refer** for non-surgical weight loss intervention (BMI 30-35 kg/m2)?
   - 0
   - 110%
   - 11-20%
   - 21-30%
   - 31-40%
   - 41-50%
   - 51-60%
   - 61-70%
   - 71-80%
   - 81-90%
   - 91-100%
3. The following statements describe your experiences with bariatric surgery. For each statement, please indicate your level of agreement.

|  | **Strongly disagree** | **Disagree** | **Neither agree nor disagree** | **Agree** | **Strongly agree** | **Do not know** |
| --- | --- | --- | --- | --- | --- | --- |
| 1. I am usually successful helping morbidly obese patients lose weight without surgery |  |  |  |  |  |  |
| 1. I feel competent prescribing weight loss programs for patients with morbid obesity |  |  |  |  |  |  |
| 1. I have extensive knowledge of bariatric surgery |  |  |  |  |  |  |
| 1. I have a good knowledge of the criteria for referral to bariatric surgery in Ontario |  |  |  |  |  |  |
| 1. I refer patients to the Ontario Bariatric Network for bariatric surgery if they meet the criteria for bariatric surgery |  |  |  |  |  |  |
| 1. I often suggest referral for bariatric surgery when I meet a patient in my clinic who is a candidate for bariatric surgery |  |  |  |  |  |  |
| 1. It is most often the patients themselves, who bring up the question of bariatric surgery. |  |  |  |  |  |  |
| 1. It is most often me who brings up the question of bariatric surgery. |  |  |  |  |  |  |
| 1. It is my impression that few obese patients seek consultations with the purpose to receive information about bariatric surgery. |  |  |  |  |  |  |
| 1. I have often consulted the Ontario Bariatric Network website when I was not sure if a patient fulfilled the criteria for bariatric surgery. |  |  |  |  |  |  |
| 1. I have often consulted the Ontario Bariatric Network website when I was uncertain about possible contraindications for bariatric surgery (e.g., psychiatric comorbidity). |  |  |  |  |  |  |
| 1. I have often consulted the Kingston Bariatric Centre of Excellence when I thought a patient was suitable for bariatric surgery, but did not meet the age criteria (age ≤ 65). |  |  |  |  |  |  |
| 1. Long term follow-up is required for patients who have undergone bariatric surgery. |  |  |  |  |  |  |
| 1. Long term follow-up should be the responsibility of the surgeon / Bariatric Centre of Excellence and not the primary care provider. |  |  |  |  |  |  |
| 1. I feel competent to address the medical complications of bariatric surgery. |  |  |  |  |  |  |
| 1. I have the resources necessary to provide good quality long-term medical care for patients who have had bariatric surgery. |  |  |  |  |  |  |
| 1. I feel comfortable with long term follow-up of patients who have had bariatric surgery. |  |  |  |  |  |  |

1. Have you ever referred patients with morbid obesity for surgical and non-surgical weight loss interventions?

- Yes
- No

b) If you answered yes to 10 (above), why do you refer patients with morbid obesity for surgical and non-surgical weight loss interventions?

*(Select all that apply)*

- Patient request
- Guideline recommendations
- Prevention of long term medical consequences of morbid obesity
- Prevention of long term psychosocial consequences of morbid obesity
- Need for additional support in management of these patients
- Ease of the referral process to Ontario Bariatric Network
- Other (Please explain)

­­­­­­­­­­­­­­­­­­­­­­­­­­­­­­­­___________________________________________________________________

___________________________________________________________________

___________________________________________________________________

___________________________________________________________________

**Part C: Reservations about Bariatric Surgery**

1. The following statements describe conditions that may influence the probability of you referring patients for bariatric surgery. For each statement, please indicate your level of agreement.

|  | **Strongly disagree** | **Disagree** | **Neither agree nor disagree** | **Agree** | **Strongly agree** | **Don’t know** |
| --- | --- | --- | --- | --- | --- | --- |
| 1. I am hesitant to refer patients for bariatric surgery |  |  |  |  |  |  |
| 1. I am concerned about the risk associated with the operation (e.g., anastomotic leakage, infection, bleeding, pulmonary embolism). |  |  |  |  |  |  |
| 1. I am concerned about postoperative surgical complications (e.g., anastomotic ulcer, internal hernia, symptomatic gallstones, chronic abdominal pain). |  |  |  |  |  |  |
| 1. I am hesitant to refer patients for bariatric surgery because I am concerned about postoperative medical complications (e.g., mineral and vitamin deficiencies, post-operative hypoglycemia, dumping syndrome). |  |  |  |  |  |  |
| 1. I am hesitant to refer patients for bariatric surgery because I am concerned about the risk of psychiatric side effects (e.g., new onset or worsening depression, substance abuse). |  |  |  |  |  |  |
| 1. I am hesitant to refer patients for bariatric surgery due to the lack of long-term data on the effects of bariatric surgery on obesity-related co-morbidities. |  |  |  |  |  |  |
| 1. I am hesitant to refer patients for bariatric surgery due to past negative experiences. |  |  |  |  |  |  |
| 1. I am hesitant to refer patients for bariatric surgery because I do not feel competent to discuss risks and benefits of surgery as a treatment option. |  |  |  |  |  |  |
| 1. I think that the internet, social medial and media coverage of bariatric surgery plays a positive role in the patients’ wish to undergo bariatric surgery. |  |  |  |  |  |  |
| 1. The Bariatric Centre of Excellence rejects patients whom I consider suitable for bariatric surgery. |  |  |  |  |  |  |

1. Do you have other concerns not listed above? (Please explain)

___________________________________________________________________

___________________________________________________________________

___________________________________________________________________

___________________________________________________________________

**Part D: Future Treatment of Severe Obesity**

1. The following statements aim to clarify your thoughts on the future treatment options for patients with morbid obesity. For each statement, please indicate how strongly you agree.

|  | **Strongly disagree** | **Disagree** | **Neither agree nor disagree** | **Agree** | **Strongly agree** | **Do not know** |
| --- | --- | --- | --- | --- | --- | --- |
| 1. The future treatment of patients with morbid obesity must be based primarily on lifestyle intervention and behavioral modification. |  |  |  |  |  |  |
| 1. The future treatment of patients with morbid obesity must be based primarily on medical management and dietary restriction. |  |  |  |  |  |  |
| 1. The future treatment of patients with morbid obesity must be based primarily on bariatric surgery with appropriate behavioral and dietary modifications. |  |  |  |  |  |  |

1. Do you have other thoughts about the future treatment options of patients with morbid obesity not listed above? (Please explain)

___________________________________________________________________

___________________________________________________________________

___________________________________________________________________

___________________________________________________________________

**Part E: Continuing Professional Development**

1. Have you participated in education on the management of patients with morbid obesity in the last five years?

- Yes
- No

1. Do you think there is a need for education for yourself or your fellow Primary Care Providers on the management of morbid obesity?
   - Y
   - N
   - Depends (Please explain) ______________________________________________________________________

______________________________________________________________________

______________________________________________________________________

______________________________________________________________________

1. How much time would you be willing to devote to learning about management of patients with morbid obesity?
   - One hour
   - Half day
   - Full day
   - Other (Please specify)

______________________________________________________________________

______________________________________________________________________

______________________________________________________________________

______________________________________________________________________

1. How important do you think it is to be knowledgeable about medical treatment options (non-surgical interventions) for patients with morbid obesity?

- Not at all important
- Somewhat important
- Neutral
- Important
- Very Important

1. How important do you think it is to be knowledgeable about surgical treatment options for patients with morbid obesity?

- Not at all important
- Somewhat important
- Neutral
- Important
- Very Important

1. Please rank your preferred methods of receiving Continuing Professional Development about management options for patients with morbid obesity:

___Suggested Readings

___Online Modules

___Seminars

___Workshops

___Grand Rounds

___Faculty Retreat

___Conferences

___Other (please specify)____________________________________________________

**Part F: Demographics**

1. What is your gender? ________________________
2. How old are you? __________________________
3. How many years have you been in practice as a Primary Care Provider? ________________
4. How is your MAIN patient/client care setting organized?

- Solo practice
- Group practice (e.g. FHO or FHN), **not including** Family Health Team
- Academic Family Health Team
- Community Family Health Team
- Community Health Centre
- Other Community Agency
- Other (please specify

1. If you work in an interprofessional practice (eg. Family Health Team), please indicate what other health care providers are a part of your practice:

*(select all that apply)*

- Nurse practitioners
- Physician assistants
- Nurses (eg. RN, RPN)
- Dietician / Nutritionist
- Occupational therapist
- Physiotherapist
- Kinesiologist / Athletic therapist
- Social worker
- Psychologist
- Pharmacist
- Medical office assistants
- Other (please specify) ____________________________________________________

1. What type of population is primarily served in your practice?
   - Rural
   - Urban
2. What is your postal code? ____________________
